# Experimental Investigation of the Cross-Sensitivity and Size Effects of Polyvinylidene Fluoride Film Sensors on Modal Testing

**DOI:** 10.3390/s121216641

**Published:** 2012-12-04

**Authors:** Kuo-Chih Chuang, Chien-Ching Ma, Hong-Cin Liou

**Affiliations:** 1 School of Aeronautics and Astronautics, Institute of Applied Mechanics, Zhejiang University, Hangzhou 310027, China; E-Mail: chuangkc@zju.edu.cn; 2 Department of Mechanical Engineering, National Taiwan University, Taipei 10617, Taiwan; E-Mail: r98522512@ntu.edu.tw

**Keywords:** PVDF film sensor, cantilever beam, cross-sensitivity, size effect, transient response

## Abstract

Due to advantages such as light weight, flexibility, and low cost, polyvinylidene fluoride (PVDF) films have been widely used in engineering applications as sensors for detecting strain, pressure, or micro-force. However, it is known that PVDF strain sensors have strain cross-sensitivity in mutually orthogonal directions. Furthermore, the size of the PVDF film sensor would also affect the dynamic strain sensing performance. In this paper, to investigate the cross-sensitivity and size effects experimentally, we employ PVDF film sensors to perform dynamic measurements on a cantilever beam. Since the vibrations of the cantilever beam are excited by impacts of a steel ball, the induced highly repeatable transient responses contain a wide range of resonant frequencies and thus can be used to investigate both the size and cross-sensitivity effects of the PVDF film sensors in a dynamic sensing environment. Based on the experimental results of the identified resonant frequencies compared with results obtained from a strain gauge, finite element calculations, and theoretical predictions, suggestions for the use of the PVDF strain sensor in modal testing are given in this paper.

## 1. Introduction

After the discovery of the high piezoelectricity in polyvinylidene fluoride (abbreviated as PVDF or PVF_2_) films by Heiji Kawai in 1969, PVDF films have been studied in research and employed in many practical applications [1]. In acoustic applications, since the acoustic impedance of the PVDF film sensor (about 3.94 × 10^6^ rayl) is close to that of water (about 1.5 × 10^6^ rayl), a PVDF film sensor can be directly used as a sonar sensor in the water without a matching layer, which is required by other piezoelectric materials such as PZTs [2]. On the other hand, PVDF film sensors can also be used to detect pulse or wave signals from the human body because the impedance of the PVDF is close to that of human body tissues. Based on this characteristic, a contact-type microphone can be developed to pick up the sound generated from vocal cords while reducing the interference of noise in the air [3]. Furthermore, PVDF film sensors have advantages such as high flexibility, extreme lightness, and high mechanical strength to endure physical impacts. Thus, PVDF film sensors have also been employed in applications such as nondestructive damage detection, force sensing, structural health monitoring, robotics, or vibration control [4–9].

To broaden their applications, in this work we employ PVDF film sensors in structural modal testing. Theoretical analysis of the use of PVDF film sensors for modal testing on a cantilever beam had been proposed by Wang *et al.*[10]. In fact, when employing PVDF film sensors, some factors such as working temperature, humidity, pyroelectric effect, or working time span might affect their sensing performances. Some researchers have investigated methods to compensate these influences in order to obtain acceptable sensing results [4,11]. Cross-sensitivity and size effects are two factors of piezoelectric sensors known theoretically to affect the sensing responses. To allow a PVF_2_ lamina to measure the strain rate only along a specified direction, innovative methods considering the surface electrode, skew angle, or polarization profile had been proposed by Lee *et al.*[12] In Lee’s work, the proposed methods are examined by comparing the sensing results obtained by PVF_2_ lamina with those obtained by differentiating the signals of a traditional strain gage. In this paper, we focus experimentally on two effects (*i.e.*, cross-sensitivity and size effect) affecting general PVDF film sensors in a situation where PVDF film sensors are used to measure strain in structural modal analysis, without considering factors such as surface electrode or skew angle. The measurement results obtained from the PVDF film sensors are compared with those obtained from a traditional strain guage. Since the signals of the traditional strain guage in this work are not differentiated to obtain strain rate responses, high frequency modes can be investigated experimentally. Modes of a cantilever beam are examined by sensing results, theoretical and finite element calculations, respectively. Since the vibrations of the cantilever beam are excited by impact loading using a steel ball, the induced highly repeatable transient responses contain a wide range of resonant frequencies and thus can be used to investigate both cross-sensitivity and size effects of the PVDF film sensors in a dynamic sensing environment.

## 2. Sensing Principle of PVDF Film Sensors

By linear piezoelectricity, the constitutive relation is expressed as [4,5]:

(1) $$T=CS-e^{T}E$$

(2) D=εE+eS

where *T* and *S* are the stress and the strain tensors; *D* and *E* are the electric displacement and the electric field vectors, respectively. The coefficient matrices *C*, *ε*, and *e* denote the stiffness, the dielectric constant, and the piezoelectric constant, respectively. Piezoelectric materials are anisotropic. Under mechanical deformations, an open-circuit output voltage is generated by [5]:

(3) $$V_{OC}=g_{3n}x_{n}t\;\;\;\;\;\;\;\;(n=1,2,3)$$

where *g*_3*n*_, *t*, and *x_n_* are the piezoelectric coefficient, the film thickness, and the stress applied in direction *n*, respectively. From Equation (3), we can see that applied stresses from mutually orthogonal directions would all contribute to the overall output voltage. Due to their internal resistance, PVDF film sensors are not suitable for static measurements.

When a PVDF film sensor is directly connected to an oscilloscope to record the transient strain responses, the voltage on the oscilloscope *V_L_* can be expressed as:

(4) $$V_{L}=V_{\mathrm{OC}}\frac{R_{L}}{R_{L}+Z_{C}}$$

where *R_L_* is the input resistance of the oscilloscope and *Z_C_* is the resistance of the PVDF film sensor. *Z_C_* equals to 1/*jωC*_0_, where *ω* is the angular velocity measured in rad/s and *C*_0_ indicates the equivalent capacitance, expressed as:

(5) $$C_{0}=\varepsilon \frac{A}{t}$$

where *A* is the electrode-covered area. To reduce the loading effect of the oscilloscope, a charge amplifier can be employed. When the PVDF film sensor is connected to the charge amplifier with a feedback capacitance *C_f_*, a feedback resistance *R_f_*, and a gain *A_C_* between the absolute value of the output voltage *V_O_* and the input voltage *V_i_* of the charge amplifier, the current *i* flowing through *C_f_* and *R_f_* can be expressed as [13]:

(6) $$\begin{array}{l} i=\left(V_{i}-V_{o}\right)\left(j\omega C_{f}+\frac{1}{R_{f}}\right)\\ \;\;\;=V_{i}\left(j\omega (A_{C}+1)C_{f}+(A_{C}+1)\frac{1}{R_{f}}\right) \end{array}$$

After applying the Kirchhoff’s current law, the output voltage can be obtained as:

(7) $$V_{O}=\frac{-j\omega A_{C}V_{S}C_{0}}{j\omega \left[\left(A_{C}+1\right)C_{f}+C_{0}+C_{C}\right]+\frac{1}{R_{a}}+\left(A_{C}+1\right)\frac{1}{R_{f}}}$$

where *R_a_* is the output impedance of the PVDF film sensor, *C_C_* is the equivalent capacitance of electric wire, and *V_S_* is the voltage generated by the PVDF film sensor [13,14].

The PVDF films employed in this paper are manufactured by Measurement Specialties, Inc. (Part number: 1-1004346-0, Hampton, VA, USA). Only the piezo constant along the drawn (*n* = 1) and thickness (*n* = 3) directions are provided by the manufacturer. The piezo stress constant of the PVDF film is *g*_31_ = 216 × 10^−3^ mV/N and *g*_33_ = −330 × 10^−3^ mV/N [15]. In the next section, based on modal testing on a cantilever beam, we will first investigate the cross-sensitivity of mutually orthogonal directions of PVDF film sensor. Then, we will discuss the size effect of the PVDF film sensor.

## 3. Natural Frequencies of the Cantilever Beam

### 3.1. Bending Mode

In this section, natural frequencies of the bending modes of a cantilever beam, in which the length of the beam is ten times larger than the width of the beam, are derived according to the Bernoulli-Euler beam theory [14]. The governing equation of motion of the cantilever beam is expressed as:

(8) $$\frac{\partial^{4}y}{\partial x^{4}}+\frac{1}{a^{2}}\frac{\partial^{2}y}{\partial t^{2}}=0$$

where *y* is the transverse displacement of the beam, and:

(9) $$a^{2}=\frac{EI}{\rho A}$$

in which *E* is Young’s modulus, *ρ* is the density, *A* is the area of the surface of the cantilever beam, and *I* is the moment of inertia. By considering the boundary conditions of the cantilever beam and the steady-state vibration problem, in which displacement expressed as *Y*(*x*)*e^iwt^*, the frequency equation can be obtained as:

(10) cosβlcoshβl=−1

where *l* is the length of the cantilever beam and:

(11) $$\beta^{4}=\omega^{2}/a^{2}$$

From Equations (10) and (11) the theoretical natural frequencies of the bending modes of the cantilever beam can be calculated as:

(12) $$f_{n}=\frac{{\left(\beta_{n}l\right)}^{2}}{2\pi l^{2}}\sqrt{\frac{EI}{\rho A}}$$

### 3.2. Torsional Mode

The governing equation of motion for the torsional modes is [14]:

(13) $$C_{T}\frac{\partial^{2}\theta }{\partial x^{2}}=\rho J\frac{\partial^{2}\theta }{\partial t^{2}}$$

where *θ* describes the angle of twist, *C_T_* is the torsional stiffness, and *J* is the polar area moment of inertia. The torsional stiffness, *C_T_*, is expressed as:

(14) $$C_{T}=\frac{cb^{3}}{3}G$$

where *c* is the width, *b* is the thickness, and *G* is the shear modulus of the cantilever beam. By considering steady-state solution *θ*(*x*,*t*) = (*A sinkx* + *B cos kx*)*e^iwt^* and the boundary condition of the beam, *k_n_* must satisfy the following condition:

(15) $$k_{n}=\frac{2n-1}{2l}\pi$$

The natural frequencies of the cantilever beam can be expressed as:

(16) $$f_{n}=\frac{\left(2n+1\right)}{2l}\frac{b}{c}\sqrt{\frac{G}{\rho }}$$

## 4. Experimental Results

**Figure 1.**
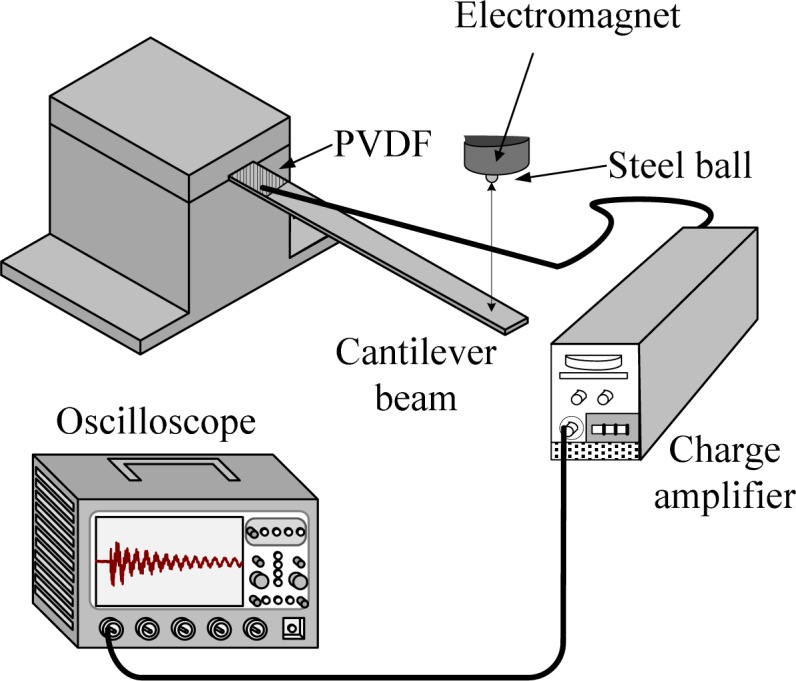
Illustration of the experimental setup.

**Figure 2.**
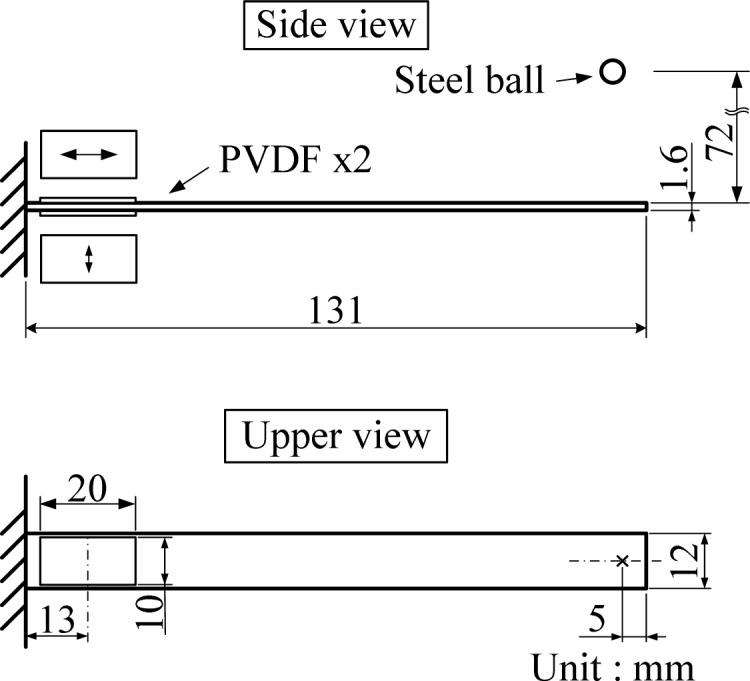
Setup of the first experiment for investigation of the cross-sensitivity.

Figures 1 and 2 illustrate the experimental setup and the dimensions of the cantilever beam used in this work, respectively. The cantilever beam is made of 1050 aluminum. Two experiments are performed on the cantilever beam to investigate the cross-sensitivity and the size effect of the PVDF film sensor.

First, cross-sensitivity of mutually orthogonal directions (*i.e.*, drawn (*n* = 1) and transverse (*n* = 2) direction) is examined. Two PVDF film sensors are bonded on the upper and bottom surfaces of the cantilever beam, respectively, to detect the transient response induced by impact loadings, where the drawn direction of the upper and bottom PVDF film sensors are respectively parallel to the axial direction and width direction of the cantilever beam. The dimensions of the PVDF film sensor are 10 mm in width, 20 mm in length, and 28 μm in thickness. For convenience, we will use “PVDF-U” and “PVDF-B” when mentioning the two sensors, where “U” and “B” indicate the upper and bottom surfaces, respectively. An impact loading is generated by freely dropping a steel ball of 4.76 mm diameter. The initial location of the steel ball is fixed by an electromagnet, 72 mm above the upper beam surface, and the impact location was 5 mm from the free end and located on the central line of the cantilever beam, as shown in Figure 2. With the impact location on the central line, the motion of the cantilever beam is theoretically dominated by bending modes rather than torsional modes or other types of vibrations, providing a simpler and systematic measuring situation to the PVDF film sensors for us to analyze and investigate the cross-sensitivity effect.

**Figure 3.**
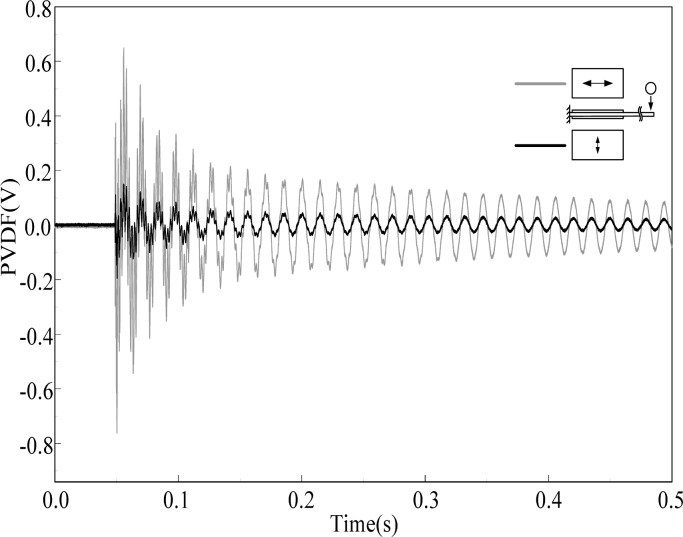
Measurement results of PVDF-U and PVDF-B.

Figure 3 shows the transient strain responses of the cantilever beam measured by PVDF-U and PVDF-B simultaneously after the steel ball is dropped at the impact location shown in Figure 2. A very high frequency oscillation can be observed at the beginning of the transient response, and both measurement results have high similarity with discrepancy of the output voltage amplitude.

**Figure 4.**
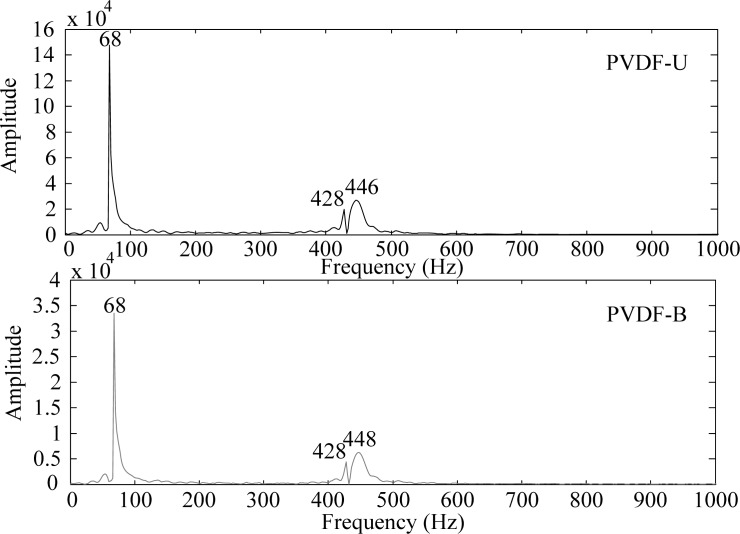
Frequency spectrum of PVDF-U and PVDF-B within 1,000 Hz.

**Figure 5.**
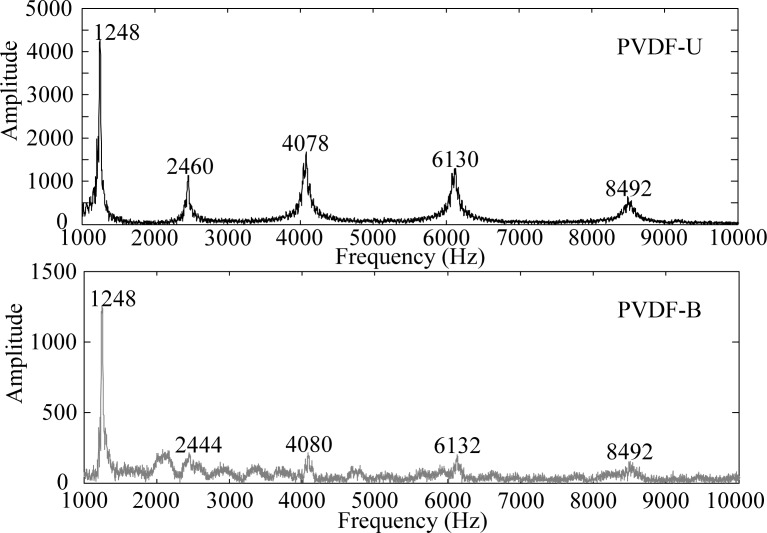
Frequency spectrum of PVDF-U and PVDF-B from 1,000 to 10,000 Hz.

**Figure 6.**
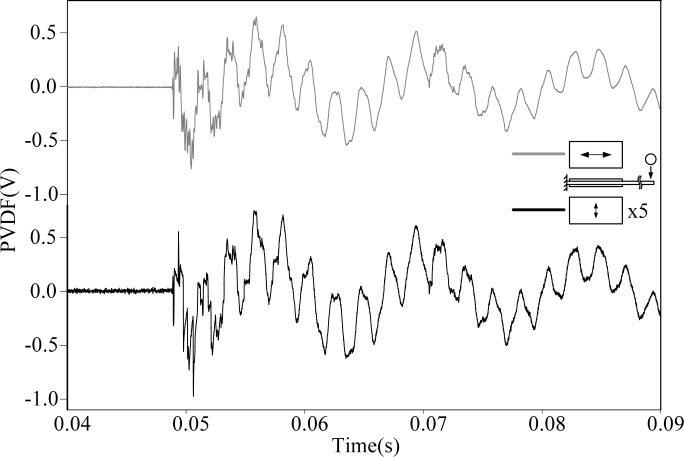
Measurement results of PVDF-U and PVDF-B with magnification.

**Figure 7.**
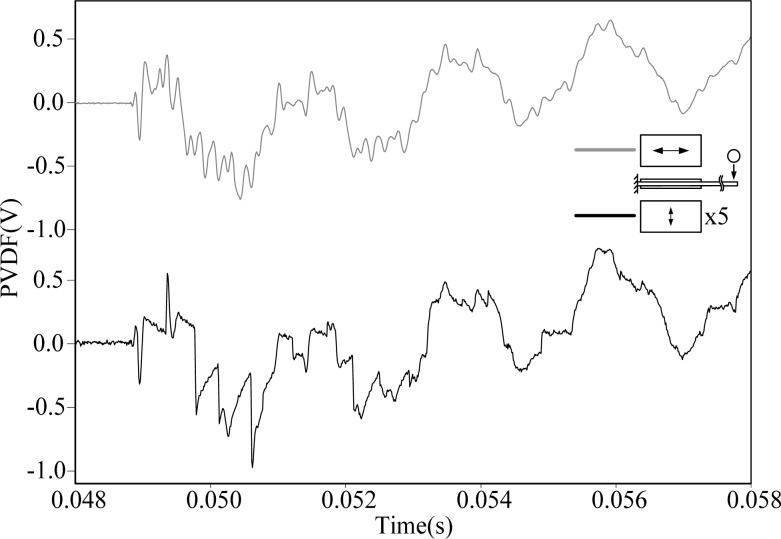
Measurement results of PVDF-U and PVDF-B with magnification within 10 ms.

By applying the Fast Fourier Transform (FFT), the time domain measurements shown in Figure 3 are transferred to the frequency domain and the results are shown in Figures 4 and 5 within 1,000 Hz and from 1,000 to 10,000 Hz, respectively. In the frequency spectra, the resonant frequencies and their corresponding spectrum amplitudes are identified. The identified corresponding amplitude of each resonant frequency is found to have an amplitude ratio of 0.2 in average (PVDF-B over PVDF-U) from the related amplitudes shown in Figures 4 and 5. Thus, we enlarge the measurement scale of PVDF-B by 5-fold and put the measurement of the PVDF-U and the 5-times-multiplied measurement results of the PVDF-B together to see the differences between the two responses, as shown in Figure 6. Figure 7 further enlarges the responses within 10 ms. From Figures 6 and 7, we can see that the sensing result obtained by the PVDF-U has more details of higher frequency oscillation at the initial transient response and less sensing noise before the oscillation has started. Although there are some minor discrepancies between the two signals (original PVDF-U and scaling PVDF-B), good agreement between them can be obtained after the initial responses (*i.e.*, 5 ms after the initial responses in Figure 7). Thus, we experimentally demonstrate that the PVDF film sensor can detect dynamic strain response in both planar mutually orthogonal directions, with higher sensitivity and the better noise performance in the drawn direction (*n* = 1). However, if noise and high frequency contents are not of concern, both directions can offer acceptable transient measuring results. We can also see that if a PVDF film sensor is employed in a two-dimensional strain field and the strain components in two directions are within the same order, the measurement obtained by the PVDF film sensor will be dominated by the signal obtained along the drawn direction.

Next, we perform the second experiment and investigate the size effect of the PVDF film sensor on modal testing. Unlike the first experiment, which focuses on measurement performances for transient responses, we will consider both transient and frequency responses in the second experiment in detail. In order to reduce the noise and enhance high frequency sensing ability, the drawn direction of the PVDF film sensor in the second experiment is along the axial direction of the cantilever beam.

**Figure 8.**
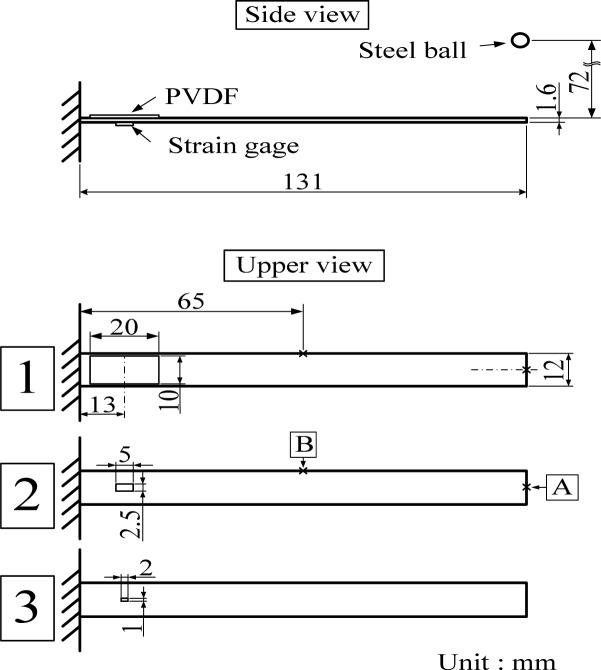
Setup of the second part experiment.

**Figure 9.**
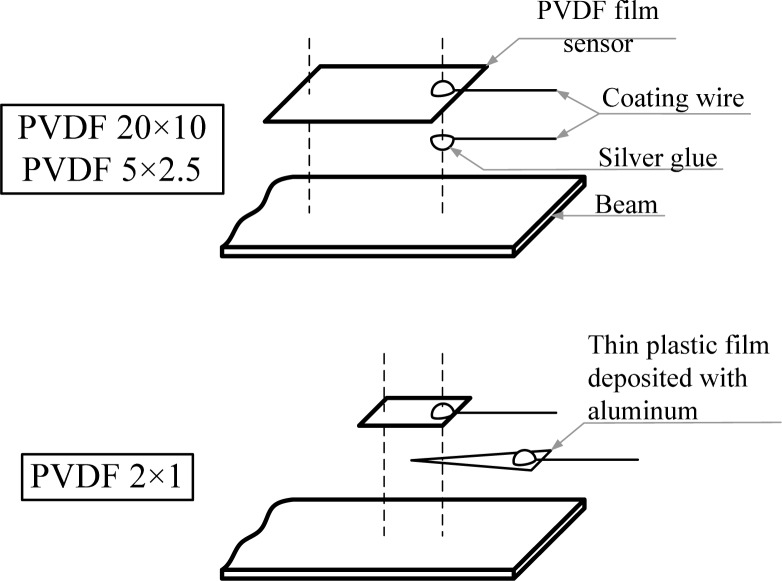
Illustration of bonding of the PVDF film sensor.

Figure 8 shows the experiment setup of the sensors on the cantilever beam. Modes of the cantilever beam are excited by impacts of the steel ball at two different locations (*i.e.*, A and B shown in Figure 8). Three PVDF film sensors with different sizes, as shown in Figure 8, are bonded on the upper surface of the cantilever beam and a strain gage (KYOWA, Tokyo, Japan, KFG-1-120-C1-11L3M2R) is bonded on the bottom surface for comparison due to its uniaxial sensing capability. For convenience, we will use PVDF 20 × 10, PVDF 5 × 2.5, and PVDF 2 × 1 as abbreviations when mentioning any of these PVDF film sensors later in this paper. The sensors are bonded with their geometric centers located along the central line of the cantilever beam and 13 mm away from the fixed end. Although the PVDF film sensor is a self-generated sensor and can be connected directly to the oscilloscope for recording measurement results, phase and amplitude of the responses might be influenced by the loading effect especially in situations of measuring low frequency modes. Hence, a charge amplifier (Signal Conditioner 2775AM4, by Endevco Corp., San Juan Capistrano, CA, USA) is employed for signal conditioning. Then, the sensing signals are recorded by the oscilloscope (Wavesurfer 64XS, LeCroy, Chestnut Ridge, NY, USA). As the size of the PVDF film sensor gets smaller, care should be taken to bond the sensor on the surface of the cantilever beam. Bonding methods in this work for the three PVDF film sensors (*i.e.*, PVDF 20 × 10, PVDF 5 × 2.5, and PVDF 2 × 1) are illustrated in Figure 9. It should be noted that before bonding the PVDF film sensor the surface of the beam should be cleaned by acetone. The PVDF film sensors are bonded on the surface of the cantilever beam with strain-guage cement (KYOWA) and the influence of the bonding layer to the sensing responses is neglected. Since the strain gage is located at the opposite surface to PVDF film sensor, the measurement results obtained by the strain guage will be inverted in this paper.

**Figure 10.**
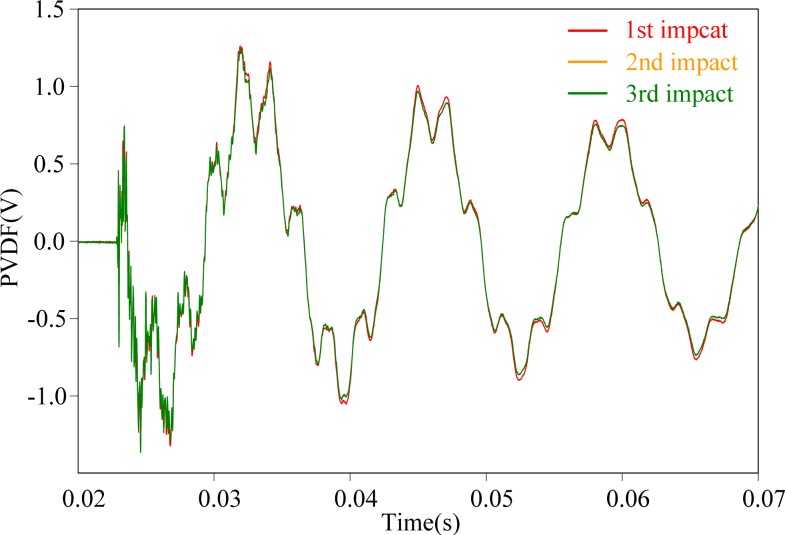
Experiment reproducibility with impact on location A.

It should be noted that we can only employ one PVDF film sensor and one strain guage to obtain the measurements simultaneously at one time. After the impact and signals detecting are done, the size of the PVDF film sensor is changed and the procedure is performed once again to obtain the measurement results. Thus, repeatability of the steel ball impacts is especially important in this work. Before performing the experiments, the steel ball is dropped three times repeatedly on point A and the signals obtained by the PVDF 20 × 10 is recorded to check the repeatability of the experimental setup. The results are shown in Figure 10 and excellent agreement can be found among these signals. From Figure 10, we can see that the impact responses induced by the steel ball are highly repeatable.

**Figure 11.**
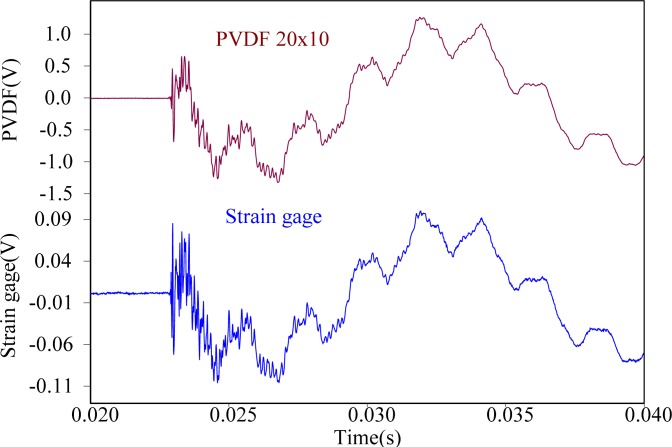
Measurement results of PVDF 20 × 10 and the strain guage with impact on location A.

**Figure 12.**
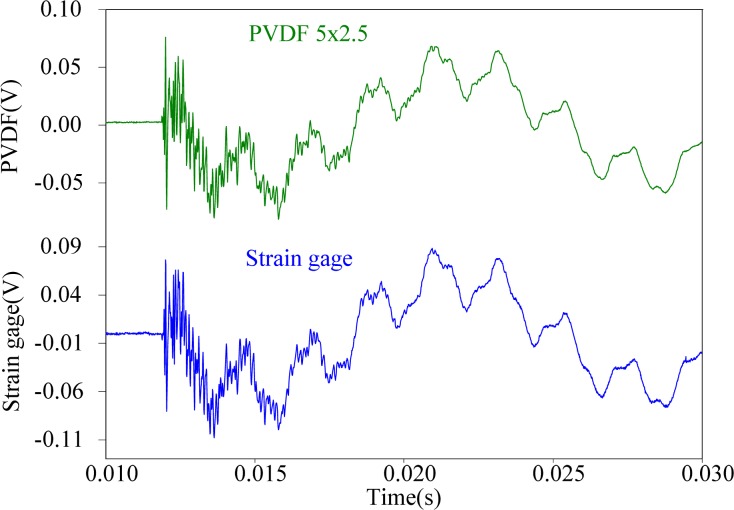
Measurement results of PVDF 5 × 2.5 and the strain guage with impact on location A.

**Figure 13.**
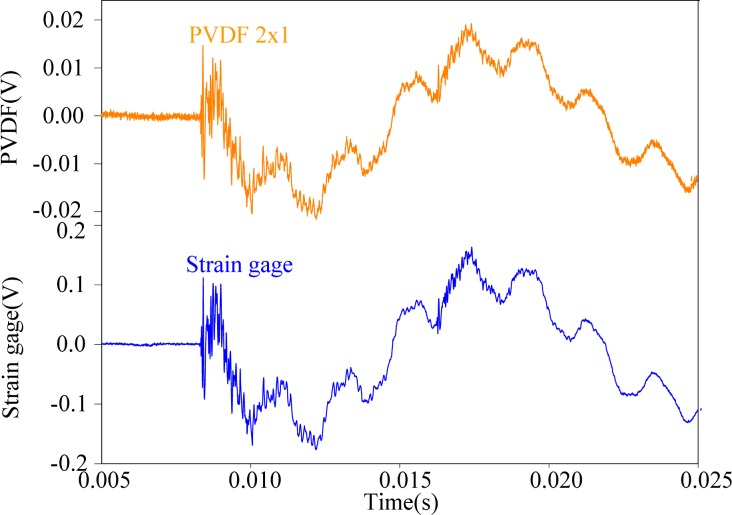
Measurement results of PVDF 2 × 1 and the strain guage with impact on location A.

First, the steel ball is dropped on the impact location A, the measuring results obtained by the PVDF film sensors are shown in Figures 11–13, respectively. All three figures show that the measurement results obtained by the PVDF film sensors and strain gage are highly consistent with each other, which indicates that the three PVDF film sensors with different sizes are able to detect the transient response correctly. Since the effective capacity of PVDF film sensor depends on its film area, the larger PVDF film sensor has larger output voltage amplitude, while the smaller sensor has less output. In other words, the measuring performance of smaller sensor has higher possibility to be affected by the electromagnetic noises and has worse signal-to-noise ratio. Hence, by comparing the three figures, we can find that the measurement result obtain by PVDF 20 × 10 has the strongest output and the best signal-to-noise ratio, due to the low output impedance of the larger size PVDF (*i.e.*, with high capacitance).

**Figure 14.**
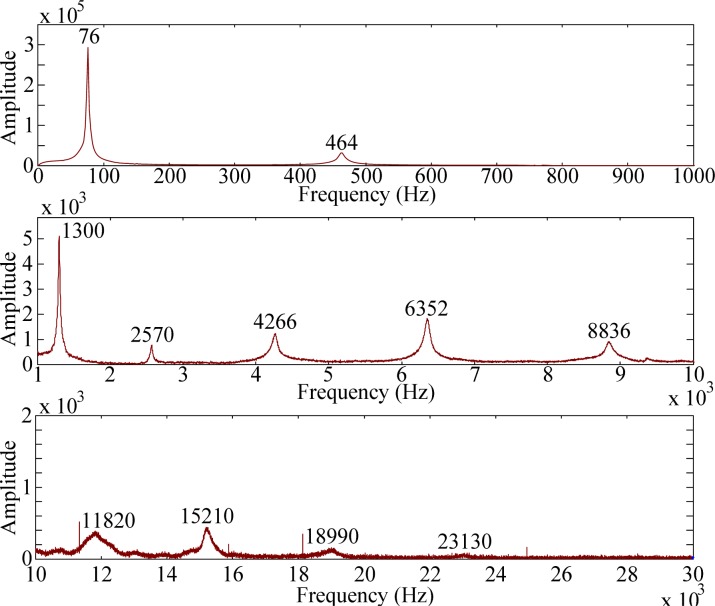
Frequency spectrum of PVDF 20 × 10 with impact on location A.

**Figure 15.**
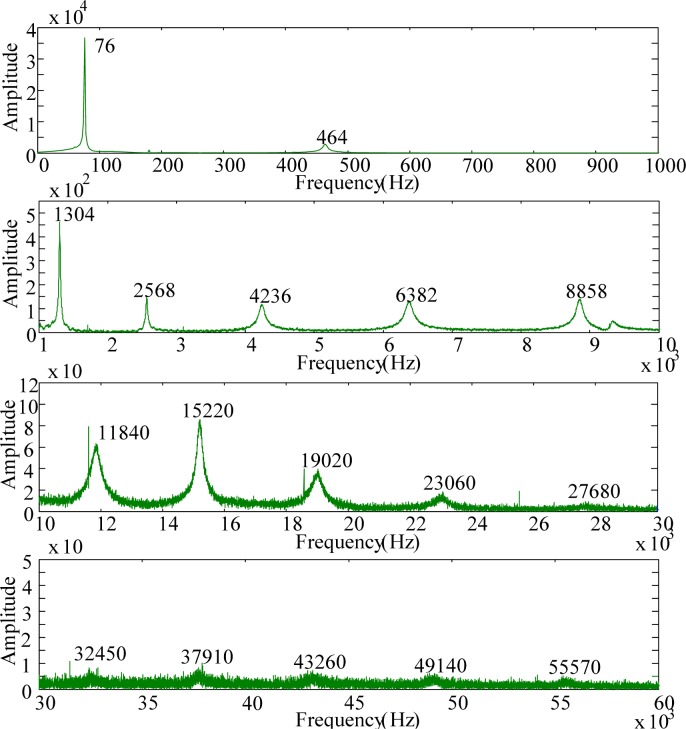
Frequency spectrum of PVDF 5 × 2.5 with impact on location A.

**Figure 16.**
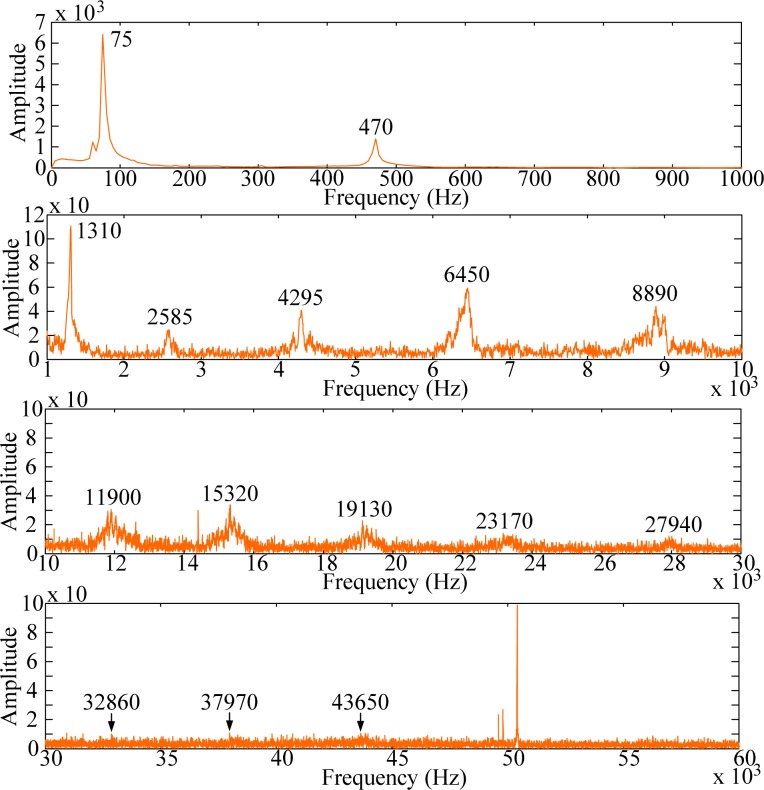
Frequency spectrum of PVDF 2 × 1 with impact on location A.

For modal testing, information in frequency domain is more important than that in time domain. By means of the FFT, the signals in time domain are transferred into frequency domain as shown in Figures 14–16. It should be noted that, to make all results clear, these results are not shown on the same scale to prevent difficulties for identification and discussion when the size of the PVDF film sensor gets smaller. Good agreement in resonant frequencies can be found among these frequency spectra. However, due to the high output impedance of the small size PVDF film, the sensing noise is observed to be more obvious in the frequency response of PVDF 2 × 1. Table 1 shows the resonant frequencies obtained by the PVDF film sensors, theoretical predictions from the Euler-Bernoulli beam theory, and finite element calculations. From Table 1 we can see that the resonant frequencies obtained by PVDF film sensors are highly consistent to those obtained from finite element calculations and the errors are less than 3.23%. Due to the average effect of the sensing area of the PVDF film sensor, larger sensor is unable to detect the higher-frequency oscillation. Hence only 11 vibration modes (*i.e.*, within 23,130 Hz) could be found in the spectrum of PVDF 20 × 10, while PVDF 5 × 2.5 and PVDF 2 × 1 are capable of detecting up to 17 and 15 modes in the spectra, respectively. The frequency spectra of PVDF 2 × 1 are quite clear and easy to identify in the interval below 30,000 Hz, but hard to distinguish above 30,000 Hz, while those of PVDF 5 × 2.5 are all clear and easy to be distinguish. Theoretically, the size of the PVDF film sensor is expected to be small enough or compatible to the smallest wave length [12]. However, our experimental results demonstrate that if the interesting mode is high (*i.e.*, in the case of small elastic wavelength or large wave number), sensing noise should also be taken into consideration to determine the proper size of the PVDF film sensor. Furthermore, due to the fact that the rotary inertia and shear deformation are not considered in the Euler-Bernoulli beam theory, it is interesting to point out that the errors of the predictions from the Euler-Bernoulli beam theory get larger as the modes get higher, as shown in Table 1, compared with FEM calculations and experimental results obtained by the PVDF film sensors.

**Figure 17.**
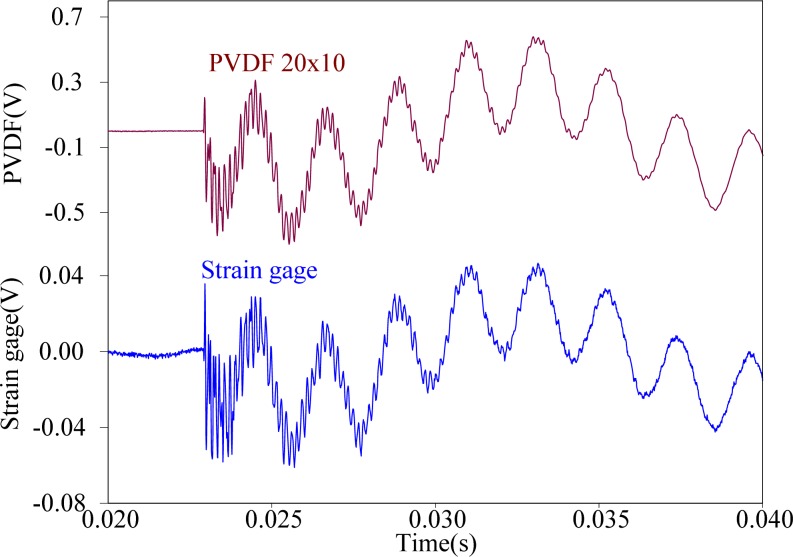
Measurement results of PVDF 20 × 10 and the strain guage with impact on location B.

**Figure 18.**
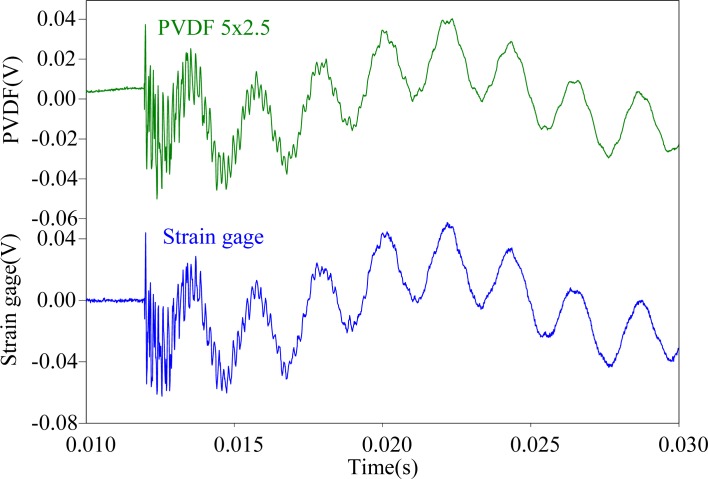
Measurement results of PVDF 5 × 2.5 and the strain guage with impact on location B.

**Figure 19.**
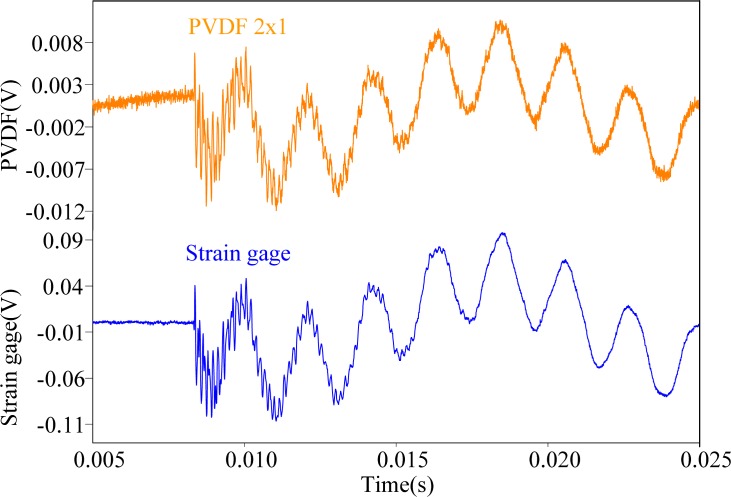
Measurement results of PVDF 2 × 1 and the strain guage with impact on location B.

Since the impact location A is on the central line of the cantilever beam, the transient responses will be dominated by the bending modes. The experiment results also agree with this prediction. Next, we change the impact location to location B that is located at the middle point of the side edge of the beam, as shown in Figure 8. At this impact location, the transient responses will theoretically contain both bending and torsional modes. Like the case of impacting on location A, we also employ three PVDF film sensors with different sizes and a strain guage as a comparison to detect the vibrating signals. The measurement results are shown in Figures 17–19, respectively. Just like the results obtained in the case of impact location A, excellent agreement between each PVDF film sensor and strain guage can also be found in the transient responses. Similarly, PVDF 20 × 10 has the strongest amplitude of output voltage and less noise, while PVDF 2 × 1 has the smallest output amplitude and is affected by electromagnetic noises most.

Table 2 shows the resonant frequencies of the torsional modes obtained by the PVDF film sensors, theoretical predictions, and finite element calculations, respectively. From Table 2, we can see that the experimental results of the resonant frequencies of the torsional modes are also highly consistent with the finite element calculations.

**Figure 20.**
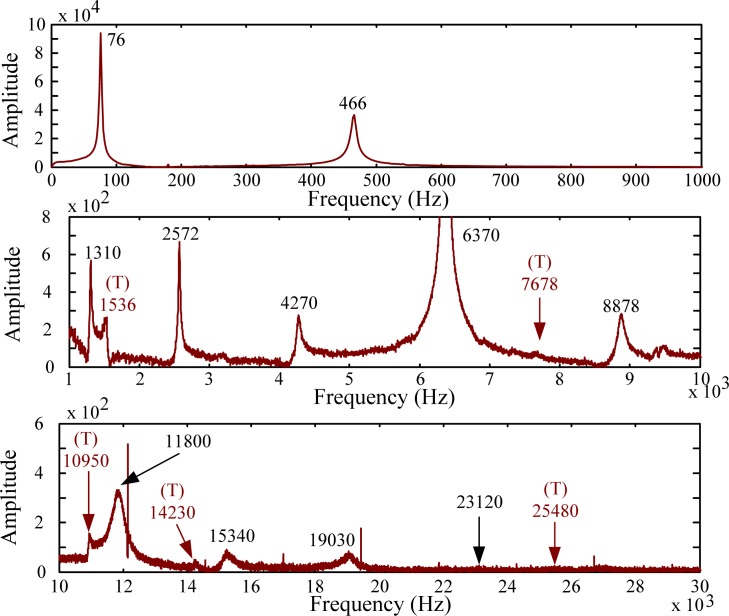
Frequency spectrum of PVDF 20 × 10 with impact on location B.

**Figure 21.**
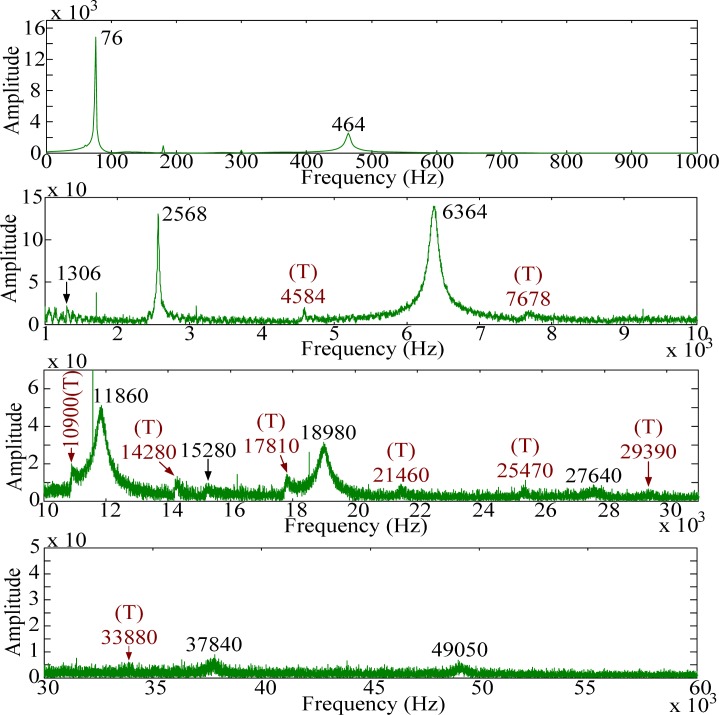
Frequency spectrum of PVDF 5 × 2.5 with impact on location B.

**Figure 22.**
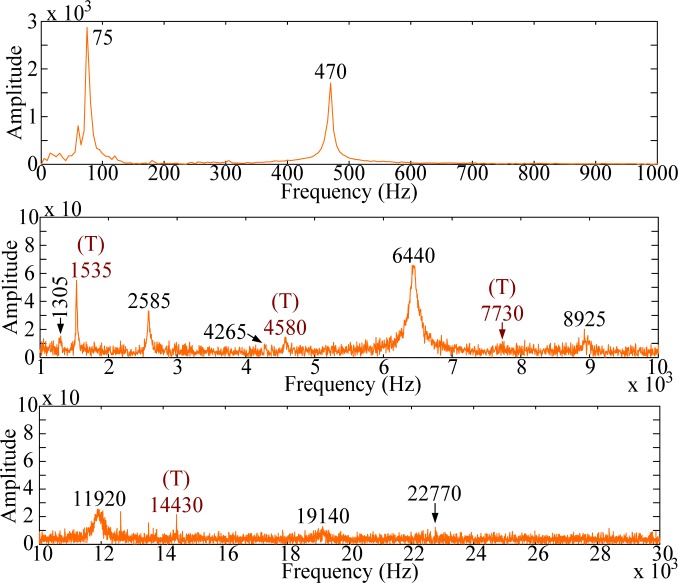
Frequency spectrum of PVDF 2 × 1 with impact on location B.

The frequency spectra are shown in Figures 20–22, respectively. As has been predicted, not only bending modes but also torsional modes are detected by the PVDF film sensors. In this case (*i.e.*, impact location B) PVDF 5–2.5 measures most torsional modes among the three PVDF film sensors. Thus, in order to have broad knowledge of the modes of the structures, different size of the PVDF film sensors should be designed as comparisons to identify or distinguish modes.

To avoid average effect, one might think it is more appropriate to use smaller sensors for modal testing. In our work, however, we show that due to the noise that influences the measurement results, PVDF film sensors with small surface area may not be the best choice for modal testing. Furthermore, although PVDF 20 × 10 (*i.e.*, larger sensing surface area) has the strongest output amplitude and best signal-to-noise performance, it is not appropriate in high-frequency measurements. For modal testing, one can at first use a small size PVDF film sensor with a buffer circuit to reduce the output impedance. Then, one can gradually enlarge the area of the PVDF film sensor to avoid the noises from affecting the identification of the interesting high frequency modes.

## 5. Conclusions

In this work, we experimentally studied the cross-sensitivity and the size effect of the PVDF film sensor and discussed sensing results on a cantilever beam subjected to impact loadings. The PVDF film sensor is able to detect the strain signal from mutually orthogonal directions simultaneously and the sensitivity is highest in the drawn direction. However, based on the experiment using the PVDF film sensor to detect the transient responses of the cantilever beam containing only bending modes, we show that the difference between the drawn and the transverse directions of the PVDF film sensor is only a amplitude ratio. Since the piezo constant along the transverse direction is usually not provided by the manufacturer, our work provides a simple but systematic way (*i.e.*, excitation of the bending modes of a cantilever beam) of considering the sensing difference between the two mutually orthogonal directions (*i.e.*, the drawn (*n* = 1) and the transverse (*n* = 2) directions).

Second, the experimental results of the size effect show that the largest PVDF film sensor (*i.e.*, PVDF 20 × 10) has the largest output voltage and signal-to-noise ratio. But it is not appropriate for high-frequency signals detecting above 30,000 Hz. The smallest sensor (*i.e.*, PVDF 2 × 1) is extremely small and can detect some high-frequency signals of the dynamic responses. However, it is subjected to the most serious noise problem due to its weak output amplitude. Compared with the above PVDF film sensors, PVDF 5 × 2.5 has the best sensing performance to identify the bending modes and torsional modes in all-frequency region for the cantilever beam used in this work. Although theoretically the size of the PVDF film sensor should be compatible with the related wavelength to detect the interesting modes, high output impedance of the small size PVDF film sensor (*i.e.*, PVDF 2 × 1 in our work) induces unwanted sensing noises. This paper demonstrates that when modal testing is of major concern, the size of the PVDF film sensors plays an important role to detect interesting high frequency modes while minimizing high frequency noises.

## Figures and Tables

**Table 1.** Resonant frequencies of the bending modes identified by PVDF (impact location A ^1^).

**Mode**	**Theory**	**FEM**	**PVDF 20X10**	**PVDF 5X2.5**	**PVDF 2X1**
1	76.067 (0.67%)	76.582	76 (0.76%)	76 (0.76%)	75 (2.07%)
2	476.71 (0.58%)	479.51	464 (3.23%)	464 (3.23%)	470 (1.98%)
3	1334.8 (0.54%)	1342	1300 (3.13%)	1304 (2.83%)	1310 (2.38%)
4	2615.7 (0.49%)	2628.7	2570 (2.23%)	2568 (2.31%)	2585 (1.66%)
5	4323.9 (0.45%)	4343.6	4266 (1.79%)	4236 (2.48%)	4295 (1.12%)
6	6459.1 (0.39%)	6484.6	6352 (2.04%)	6382 (1.58%)	6450 (0.53%)
7	9021.4 (0.30%)	9048.1	8836 (2.34%)	8858 (2.10%)	8890 (1.75%)
8	12011 (0.15%)	12029	11820 (1.74%)	11840 (1.57%)	11900 (1.07%)
9	15427 (0.05%)	15420	15210 (1.36%)	15220 (1.30%)	15320 (0.65%)
10	19271 (0.30%)	19214	18990 (1.17%)	19020 (1.01%)	19130 (0.44%)
11	23541 (0.60%)	23400	23130 (1.15%)	23060 (1.45%)	23170 (0.98%)
12	28239 (0.97%)	27968		27680 (1.03%)	27940 (0.10%)
13	33363 (1.38%)	32908		32450 (1.39%)	32860 (0.15%)
14	38915 (1.86%)	38206		37910 (0.77%)	37970 (0.62%)
15	44894 (2.39%)	43847		43260 (1.34%)	43650 (0.45%)
16	51299 (3.01%)	49802		49140 (1.33%)	
17	58132 (4.07%)	55859		55570 (0.52%)	

1 Percentages are errors compared with FEM calculations.

**Table 2.** Resonant frequencies of the torsional modes identified by PVDF (impact location B ^1^).

**Mode**	**Theory**	**FEM**	**PVDF 20X10**	**PVDF 5X2.5**	**PVDF 2X1**
1	1575.9	1529.5	1536 (0.42%)		1535 (0.36%)
2	4727.8	4608.4		4584 (0.53%)	4580 (0.62)
3	7879.7	7746.5	7678 (0.88%)	7678 (0.88)	7730 (0.21%)
4	11032	10981	10950 (0.28%)	10900 (0.74%)	
5	14183	14346	14230 (0.81%)	14280 (0.46%)	14430 (0.59%)
6	17335	17873		17810 (0.35%)	
7	20487	21587		21460 (0.59%)	
8	23639	25511	25480 (0.12%)	25470 (0.16%)	
9	26791	29665		29390 (0.93%)	
10	29942	34064		33880 (0.54%)	

1 Percentages are errors compared with FEM calculations.
